# Effects of Different Growth Stages of Amaranth Silage on the Rumen Degradation of Dairy Cows

**DOI:** 10.3390/ani9100793

**Published:** 2019-10-12

**Authors:** Jian Ma, Guoqing Sun, Ali Mujtaba Shah, Xue Fan, Shengli Li, Xiong Yu

**Affiliations:** 1 College of Animal Science, Xinjiang Agricultural University, Urumchi 100193, China; Crazyma0411@163.com (J.M.); jackjeons123@163.com (G.S.); fanxue1205@163.com (X.F.); 2 Animal Nutrition Institute, Sichuan Agricultural University, Chengdu 611130, China; alimujtabashah@sbbuvas.edu.pk; 3 College of Animal Science, China Agricultural University, Beijing 830052, China; 4 Department of Livestock Production, Shaheed Benazir Bhutto University of Veterinary and Animal Sciences, Sakrand 67210, Pakistan

**Keywords:** growth stage, amaranth silage, chemical composition, effective degradability, correlation

## Abstract

**Simple Summary:**

The production of some crops is limited in parts of the world because of the shortage of water resources. Therefore, it is an irresistible trend to make full use of roughage resources. Amaranth is one of the crops which can grow in the poor soils and areas with high temperature and limited rainfall. In addition, the quality and yield of forage depend on harvest stage, and the appropriate growth stage is an important influencing factor of forage quality. The purpose of this study was to investigate the rumen degradation characteristic of amaranth silage including four kinds of growing stages. The results showed that with the extension of the growing period, the crude protein contents decreased gradually, and the contents of dry matter, neutral detergent fiber, and acid detergent fiber exhibited an opposite trend. Besides, the ruminal dry matter, crude protein, neutral detergent fiber, and acid detergent fiber degradability was significantly affected by growth stage. Our result suggested that amaranth silage can be used as a roughage in the diet of dairy cow. However, growth stage is an essential factor to influence the ruminal degradability, thus the growth stage of amaranth should be taken into consideration when making silage.

**Abstract:**

The purpose of this study was to investigate the rumen degradation characteristic of amaranth silage (*Amaranthus hypochondriacus*) including four kinds of growing stages (budding stage (BS), 50 d after planting (DAP); early flowering stage (ES), 58 DAP; peak flowering stage (PS), 70 DAP; heading stage (HS), 90 DAP). Four Holstein dairy cows with permanent ruminal cannulas were used as experimental animals. Nylon-bag method was used to assess the ruminal degradability of dry matter (DM), crude protein (CP), neutral detergent fiber (NDF), and acid detergent fiber (ADF). The results showed that the concentration of DM in HS was significantly higher than other stages (*p* < 0.05), whereas the contents of CP, were lower than in other stages (*p* < 0.05). With the extension of the growing period, the DM effective degradability of amaranth silage decreased gradually, and the difference was significant (*p* < 0.05). The ruminal CP degradation of 72 h was more than 80%, and compared with ES and HS, the degradation rate of BS and PS was significantly increased (*p* < 0.05). Compared to BS, the effective degradability of CP was increased (*p* < 0.05) in ES and HS. For ruminal NDF degradability, the effective degradability of HS was minimum, and it had a noticeable difference with BS and ES (*p* < 0.05). Thus, the different quality of amaranth growth stage including chemical contents and rumen degradation should be taken into consideration when making amaranth silage. In the present study, the optimal growth stage of amaranth was from the peak flowering stage to heading stage for ensiling.

## 1. Introduction

Roughages usually account for 40–70% in the diet of dairy cows and provide most nutrients for dairy cows [1]. Silage is a common roughage source. However, the production of some crops, such as corn, is limited in parts of the world because of the shortage of water resources [2]. Therefore, it is an irresistible trend to make full use of roughage resources, and the application of non-conventional feed resource with high crude protein (CP) concentration, digestibility, and yield and reduced water requirements as alternative feeds of dairy cows has attracted increasing attention.

Recently, researchers found that some unusual crops adapted to water scarcity and poor soils can be useful as a feedstuff for dairy cows under certain harsh conditions [3,4]. Amaranth (*Amaranthus hypochondriacus*) is one of such crops which can grow in the poor soils and areas with high temperature and limited rainfall [5]. It is a C_4_ dicotyledonous crop and is characterized by its high yield performance as well as high nutritional value [6]. According to the report, the yield of amaranth is reached up to 86.4 t fresh weight/ha and 13.2 t/ha dry matter (DM)/ha [7]. In addition, compared with corn, amaranth has a higher CP contents (up to 285 g/kg DM), a lower lignin concentration (40 g/kg DM) [8], and a low nitrate and oxalic acid concentrations [9]. In northern China, when it was harvested in the mature stage, the yield of fresh amaranth and DM was reached up to 130 t/ha and 20 t/ha, respectively. Besides, the contents of CP were about 14% during the harvest at the budding stage [10]. The above advantages make amaranth to be used as a useful forage for ruminants, and the utilization of amaranth silage in diets of fattening lambs [11] and lactating Holstein cows [3] does not change animal health and performance.

The quality and yield of forage depend on harvest stage [12]. Previous studies found that plant maturity affects the contents of DM, CP, neutral detergent fiber (NDF), and acid detergent fiber (ADF) [13]. When forage is harvested earlier, the yield of herbage is low, and the moisture contents are high, which is not suitable for ensiling. However, when the forage is harvested too late, its nutrient contents and feed value decreased [14]. Therefore, the appropriate growth stage is an important influencing factor of forage quality. In addition, an essential index of ruminant feed nutritional value evaluation is the degradation characteristics of nutrients in the rumen [15]. Nylon bag technology is the primary method to evaluate the degradation rate of ruminant feedstuff at present [16]. To our best knowledge, there is scarce information on the effects of growth stage on the ruminal degradability of amaranth silage. Therefore, this study was conducted to evaluate the changes in rumen degradation characteristics during the growth of amaranth silage.

## 2. Materials and Methods

### 2.1. Ethics Statement

The animal experiment was performed according to the Regulation on the Administration of Laboratory Animals (2017 Revision) promulgated by Decree No. 676 of the State Council. All procedures involving animal care and management were in accordance with and approved by the Institutional Animal Care and Use Committee of China Agricultural University (Beijing, China; Code No. AW09059102-2).

### 2.2. Preparation of Amaranth Silage

A field experiment was conducted during 2018 at the experimental field station (covers 800 m^2^) of Xinjiang Agriculture University in Heshuo County, Bayingolin Mongol Autonomous Prefecture (Xinjiang, China), with the latitude and longitude of 88°40′ N and 41°25′ E. The area is at an altitude of 2217 m above sea level, with a mean annual precipitation and temperature of 58.6 mm and 11.4 °C, respectively. The soil at the experimental field was soft loam-sandy, and pH was 7.83, containing 1.96% organic matter, 0.65 g/kg total N and 0.77 g/kg total K. During the growth period (May to October) of amaranth in 2018, the rainfall was 78.3 mm. The amaranth field was fertilized with urea (400 kg/ha) and potassium fertilizers (60 kg/ha). All nitrogenous and potassium fertilizers were applied as a single time before sowing as base fertilizers. The nitrogenous fertilizers were commercially available urea (N content: 46%), and potassium fertilizers were potassium chloride (K_2_ O content: 62%).

The seeds of amaranth were provided by the Chinese Academy of Agricultural Sciences and the seeding rate was about 0.8 kg/ha. A randomized complete block design was used with 4 blocks, each block contains 4 plots (9.5 m × 4.5 m), with about 1 m walkway in between. The forage was sown manually on the 15 th of May (2018), harvested at 4 growth stages (budding stage (BS), 50 d after planting (DAP); early flowering stage (ES), 58 DAP; peak flowering stage (PS), 70 DAP; heading stage (HS), 90 DAP) by cutting the whole-plants with knife to a 5-cm stubble height, and chopped into fragments of 3 cm in length using a forage chopper (model 680, Juancheng Mechanical Equipment Co., Guangzhou, China) before making ensilage. DM contents of 30–35% are usually recommended to obtain amaranth silages of good quality [10]. However, in order to reduce the influence of other factors (e.g., weather), we used the fresh amaranth, and all the silages were processed in the same method without other treatments except for chopping. Fresh amaranth at different stages was tightly compacted and sealed in a fermentation container (2 L capacity) to make silage (Supplementary Figure S1). Four replicates were set in each treatment group. The silos were stored in the laboratory at 25 ± 2 °C. After 60 d of storage, the silos were opened, and samples were collected for measurement of nutrient concentrations and nutrient rumen degradability.

### 2.3. Chemical Analysis

Samples of silage with different growth stages were collected and analyzed for nutrient composition. The fresh samples were weighed, in a forced-air oven, at 65 °C for 48 h to a constant weight to analyze the DM contents, then smashed to pass through a 1-mm sieve (Aizela electric appliance co. LTD., Zhejiang, China), and were analyzed for CP (method 984.13) using the procedures of AOAC International [17]. The concentrations of NDF and ADF in feed samples were analyzed according to Van Soest [18]. The solubilization of cellulose with sulphuric acid was used for lignin (ADL) contents (method 973.18) [17].

### 2.4. In Situ Nutrient Degradability

Four Chinese Holstein cows (body weight: 515.8 ± 12.6 kg; dry cow) with a ruminal cannula were used to measure the ruminal degradability of DM, CP, NDF, and ADF by nylon bag method [19]. Animals were cared according to the Chinese Standards for the use and care of research animals. These cows were fed 40:60 concentrate to roughage ration (DM contents was 46%) containing alfalfa, amaranth silage, Chinese wildrye, corn, soybean meal, and mineral-vitamin premix, at 0800 and 1600 h, the diet and water were provided *ad libitum* throughout the trial. Cows were adapted to the diet for 20 days. The bag size was 8 × 12 cm with a pore size of 50 μm (Ruitong Biotech Co., Ltd., Xinjiang, China). The samples were milled through a 4-mm sieve. Five grams of each sample was weighed and sealed in each nylon bag. The nylon bag containing the samples was fixed on the soft rubber stopper and then placed into the nylon net (about 50 cm in length). Nylon net was put into the rumen 1 h before morning feeding, and the other end of the net was fixed on the ruminal fistula of cows. Each sample was divided into 4 parts and put into the rumen of 4 cows. The feed samples at each time point were set as 4 replicates. The samples were incubated in the rumen for 4, 8, 16, 24, 36, 48 and 72 h.

Upon removal from the rumen, nylon bags were rinsed immediately under cold tap-water with subsequent washing in a tub with tap-water until the rinse water was clear. And then the bags were oven-dried at 65 °C to a constant weight. Residues were weighed, and ground using a ball mill to pass 1-mm screen, thoroughly mixed and analyzed for nutrient composition (DM, CP, and NDF, ADF) as previously described. The degradability value at time 0 was obtained by rinsing 4 bags per sample. The percentage of ruminal DM, CP, NDF, and ADF degradability (P) at time (t) was estimated from an exponential curve as P = a + b (1 − e^−ct^) which was fitted to the data by iterative regression analysis [20]. Then, the effective degradability (ED) of DM, CP, NDF, and ADF was calculated as ED (%) = a + b × c/(k + c), according to Øskov and McDonald [20]. In these equations, “e” is the base of natural logarithms, “a” is the soluble and very rapidly degradable fraction, and “b” is the insoluble but potentially degradable fraction which degrades at a constant fractional rate (c) per unit time (t), ED is the effective degradability, and “k” refers to the fractional outflow rate from the rumen. An assumed value for “k” was 3.1%/h [21]. NLIN program in SAS (version 9.4; SAS Institute, Inc., Cary, NC, USA) was used to calculate the values of a, b, and c.

### 2.5. Statistical Analysis

Data were analyzed by one-way ANOVA procedure of the SPSS statistical software (Ver. 20.0 for Windows; SPSS, Chicago, IL, USA). The statistically significant differences were determined by Duncan’s multiple range tests. Orthogonal polynomial contrasts were employed to detect linear and quadratic effects of advancing growth stages. The correlation between effective degradability and rumen degradability at different time points was analyzed by using Pearson correlation analysis in SPSS statistical software, and if *p* < 0.05, the linear regression analysis was used to obtain regression equations. Data were presented as mean and SEM. The significance level was indicated at *p* < 0.05, with trend was declared at 0.05 ≤ *p* < 0.10.

## 3. Results

### 3.1. Chemical Composition

The chemical compositions of amaranth silage are shown in Table 1. The results showed that the nutrient contents of silage produced by different growth stages of grain amaranth were different. The concentration of DM in HS was significantly higher than other stages (*p* < 0.05), and with the extension of the growth stage, the DM contents increased gradually, and the concentrations of NDF, ADF, and ADL showed a similar trend. In BS, the concentration of CP was highest, but it was similar to other stages (*p* > 0.05) except for HS (*p* < 0.05).

### 3.2. Ruminal DM Degradation

The rumen degradability and degradation parameters of DM are presented in Table 2. Compared with other stages, the ruminal DM degradation of 72 h in BS was highest (*p* < 0.05). All the amaranth silages degraded faster before 24 h and then leveled off. The rapidly degradable fraction of BS was higher than other stages (*p* < 0.05), and no difference was found between PS and HS (*p* > 0.05). We found a similar trend in slowly degradable fraction. With the extension of the growing period, the effective degradability of amaranth silage decreased gradually, and the differences were significant (*p* < 0.05).

### 3.3. Ruminal CP Degradation

The rumen degradability and degradation parameters of CP are presented in Table 3. The ruminal CP degradation of 24 h was similar (about 70%, *p* > 0.05) in four growth stages. Besides, in four silages, the degradation rate of CP at 72 h was more than 80%. The degradation speed of CP exhibited a similar trend with DM, and it occurred mainly before 24 h. The rapidly degradable fraction of BS was significantly lower than other stages (*p* < 0.05), interestingly, an opposite trend was found in slowly degradable fraction. The effective degradability of all silage was more than 65%, and compared with BS, the effective degradability was increased (*p* < 0.05) in ES and HS.

### 3.4. Ruminal NDF Degradation

In all growth stages, the rumen degradability of NDF at 4 h and 36 h did not have a noticeable difference (*p* > 0.05) (Table 4). Inconsistent with DM and CP, all the amaranth silages degraded slower before 16 h. The rapidly degradable fraction of four growth stages was meager, and it had no noticeable difference (*p* > 0.05). However, compared with other stages, the slowly degradable fraction of BS was significantly increased (*p* < 0.05). Compared with DM and CP, the effective degradability of NDF was lower. The effective degradability of HS was minimum, and it had a noticeable difference compared to BS and ES (*p* < 0.05).

### 3.5. Ruminal ADF Degradation

The rumen degradability and degradation parameters of ADF are shown in Table 5. We found that the ruminal ADF degradation of 4 h was slow, and the maximum was only 10.60% (HS). Consistent with NDF, all the amaranth silage degraded slower before 16 h, and from 16 h to 48 h, the degradation speed of all stages became faster. The rapidly degradable fraction of ES was minimum and significantly lower (*p* < 0.05) than PS and HS. For slowly degradable fraction, the BS was maximum and markedly higher (*p* < 0.05) than other stages. A similar trend was found in effective degradability, and the effective degradability of BS exhibited higher (*p* < 0.05) value than other silages.

### 3.6. The Correlation between Effective Degradability and Rumen Degradability at Different Time points

The correlation analysis of effective degradability and ruminal degradability at different time point are presented in Table 6. We found that the correlation of DM between the effective degradation rate and the degradation rate at 4, 24, 36, and 48 h was more than 0.9, among which the correlation at 36 h was maximum. The correlation of CP between effective degradability and ruminal degradability at 24 h was highest. However, for NDF, it did not have an obvious correlation. Compared with other time points, the correlation of ADF at 72 h was maximum.

## 4. Discussion

### 4.1. Chemical Composition

Similar to Johnson et al. [22] and Fageria et al. [23], in this experiment, plant maturity increased DM contents. Compared to other growth stages, the increasing DM contents of the amaranth silage at HS could be connected to the grain development in the ear and carbohydrates deposition in the grain [24]. In the present study, with the extension of the growth period, the CP contents of amaranth silage decreased gradually. Similar to the results of Yu et al. [25] and Sarmadi et al. [26], they noticed that as forages reached maturity, the CP contents are decreased. The reduction of CP contents with plant maturity is associated to a decrease of leaves and an increase of stems in the forage biomass, and increased lignification leads to relative decrease of CP [27]. On the other hand, the different trend was found in the concentration of NDF and ADF in this experiment. The increases of NDF and ADF contents as amaranth advanced in growth stage were possibly due to the increase stem to leaf ratio and a higher demand for fibrous tissues to maintain plant structure as grows, and compared with leaves, the cell wall components are higher in stems [28]. In addition, compared with corn silage [1], the contents of CP were higher in amaranth silage (corn silage: 8–11% VS. amaranth silage: 11.5–13.2%). However, the NDF concentration of corn silage was 46–57%, and it ranged from 47.8% to 54.1% in amaranth silage. Besides, it has been well documented that the purpose of ensiling is to reduce the pH to below 4.2, and preferably to below 4 [29]. According to our (it has submitted but not published) data, the pH of BS and ES was more than 4 (4.65 and 4.01, respectively), and higher than PS and HS (3.86 and 3.74, respectively). The silages of PS and HS showed higher lactic acid but lower acetic acid contents than BS and ES (lactic acid: 4.33 and 5.13 VS. 2.75 and 3.50, acetic acid: 1.64 and 0.99 VS. 1.90 and 1.95, respectively). These data proved that the fermentation quality of PS and HS was better than BS and ES. Even though the nutritional contents decreased with the advancing amaranth growth stage, we suggest that the amaranth growth stage from PS to HS is selected for ensiling considering the fermentation quality.

### 4.2. Ruminal DM Degradation

Dry matter intake (DMI) is essential for dairy cows to maintain health and production performance. A critical factor affecting the DMI of dairy cows is the ruminal DM degradability, which is positively correlated with DMI [30]. In this experiment, the DM degradation rate of four growth stages increased gradually with the extension of retention time in the rumen, but the increase rate was different. The rumen degradability of BS was highest, and a similar trend was found in some degradation parameters including “a”, “b”, a + b, and ED. The declining fractions a and b as the amaranth advanced in growth stage were related to a decrease of CP and an increase of fibrous components, which resulted in decrease of total degradable fraction (a + b) [28]. Previous studies have shown that the ED decreased with the increase in plant maturity [26,31]. The lower ED of HS as compared to ES was possibly due to the increase of cell wall components, especially lignin, which has negative influence on DM degradability because higher lignification decreases the colonization of bacteria and degradation [32]. In addition, in the present study, the ED of HS was minimum; however, it was higher than the ED of whole corn silage in the study of Liu et al. [33].

### 4.3. Ruminal CP Degradation

The degradation rate of CP is affected by the fermentation degree of the roughage and the retention time in the rumen. Besides, it is also affected by the true protein contents and composition proportion of CP in the roughages [34]. In this study, at 24 h, the CP degradability of all silages was about 70%, suggesting that the degradation of CP of amaranth silage was mainly before 24 h. It was consistent with the previous study about alfalfa silage [35]. Compared with the results from Liu et al. [33], at 72 h, the CP degradability of four stages was higher than whole corn silage. Satter [36] reported that higher contents of CP in roughage were beneficial for CP degrading in the rumen. Inconsistent with that report, in our study, the CP contents of BS were higher than other stages, but the ED of BS was minimum. It was possibly due to the different forage variety. In addition, the rapidly degradable fraction of BS was also minimum, which may be another reason, but this still needs further study.

### 4.4. Ruminal NDF and ADF Degradation

The crude fiber contents in roughages are essential for the rumen health of dairy cows [1]. Degradation of NDF and ADF in the rumen is an important indicator to assess the quality of roughages. Higher degradation rate of NDF and ADF is beneficial for rumen fermentation, and it can lead to higher concentrations of volatile fatty acids that can provide more energy for dairy cows, and then it can promote the production performance of dairy cows [5]. The previous study found that with the advanced growth stage the fibrous contents increased, which result in the decrease of ruminal NDF and ADF degradability [26]. However, in our study, we did not found a noticeable trend in NDF degradability. When comparing the direction of movement, the degradation of NDF and ADF in four silage of amaranth mainly occurred after 16 h. The ED of BS was significantly higher than other stages, this was due to the digestible components of amaranth decreased with the plant maturity, and it is consistent with the result from Yu et al. [25].

### 4.5. The Correlation between Effective Degradability and Rumen Degradability at Different Time points

Using different time points to evaluate the rumen degradation of roughage is a common method. However, the process takes a long time and a larger workforce. Relevant studies have confirmed that feed samples cultured in the rumen for 48 h were affected by dilution rate, and only a small amount of DM remained in the rumen [37]. Liu et al. [38] reported that the speed of starch rumen degradation rate of silage decreased after silage was cultured in the rumen of dairy cows for some time, therefore, it was speculated that there must have a specific time point that most of the starch was degraded by rumen microorganism. In our study, the results showed that the correlation of DM between the effective degradability and the ruminal degradation rate at 36 h was highest, and the correlation of CP between effective degradability and ruminal degradability at 24 h was highest. Therefore, we can speculate that it was feasible to evaluate the DM and CP ruminal degradation rate of amaranth silage in the rumen by using 24 h and 36 h degradation rate. However, because there are few studies in this research area, so more experiments should be conducted to verify it.

## 5. Conclusions

The results from our study implied that the advancing amaranth growth stage increased the contents of DM, NDF, and ADF, but deceased CP contents and the ED of DM and CP. The degradation of DM and CP mainly occurred before 24 h; however, the degradation of NDF and ADF mainly occurred after 16 h. The correlation of DM and CP between effective degradability and ruminal degradability at 36 h and 24 h was highest, respectively. Thus, the different quality of amaranth growth stage including chemical content and rumen degradation needed to be considered when making amaranth silage. In the present study, the optimal growth stage of amaranth was from the peak flowering stage to heading stage for ensiling combined with fermentation quality of silage.

## Figures and Tables

**Table 1:** Chemical composition of amaranth silage. (%, DM basis).

Items	Growth Stage				SEM	*p*-Value	
	BS	ES	PS	HS		Linear	Quadratic
DM	15.31 ^c^	17.19 ^b,c^	18.82 ^b^	22.20 ^a^	0.323	<0.001	0.198
CP	13.21 ^a^	12.52 ^a^	12.41 ^a^	11.51 ^b^	0.113	<0.001	0.028
NDF	47.88 ^b^	50.85 ^a,b^	53.39 ^a^	54.14 ^a^	1.585	<0.001	0.361
ADF	29.79 ^b^	31.03 ^b^	33.84 ^a,b^	38.03 ^a^	1.077	0.013	0.453
ADL	2.53 ^c^	2.62 ^c^	3.39 ^b^	4.67 ^a^	0.023	<0.001	0.560

BS = budding stage; ES = early flowering stage; PS = peak flowering stage; HS = heading stage. DM = dry matter; CP = crude protein; NDF = neutral detergent fiber; ADF = acid detergent fiber; ADL = lignin. In the same column, values with different letter mean significant difference (*p* < 0.05).

**Table 2:** Rumen degradability and degradation parameters of DM of amaranth silage.

Items	Growth Stage				SEM	*p*-Value	
	BS	ES	PS	HS		Linear	Quadratic
Time (%)							
4	38.89 ^a^	39.26 ^a^	36.06 ^b^	34.97 ^b,c^	0.505	0.031	0.024
8	44.34 ^b^	45.76 ^a^	45.96 ^a^	46.15 ^a^	0.838	0.043	0.367
16	53.41 ^b^	54.04 ^a^	53.24 ^b^	51.73 ^c^	1.337	0.022	0.038
24	62.01 ^a^	61.44 ^b^	59.10 ^c^	57.05 ^d^	1.287	<0.001	0.816
36	65.82 ^a^	65.36 ^a^	60.88 ^b^	60.82 ^b^	1.493	0.014	0.227
48	71.34 ^a^	66.73 ^b^	62.36 ^c^	62.56 ^c^	2.149	0.015	0.040
72	76.07 ^a^	69.78 ^b^	67.63 ^c^	66.23 ^d^	2.391	<0.001	0.711
Rumen Degradation Parameters							
a (%)	33.89 ^a^	31.07 ^b^	28.20 ^c^	28.23 ^c^	0.307	0.013	0.038
b (%)	46.23 ^a^	38.85 ^b^	37.32 ^b,c^	36.85 ^c^	0.301	0.007	0.207
a + b (%)	80.12 ^a^	69.92 ^b^	65.52 ^c^	65.08 ^c^	0.558	<0.001	0.188
c (%/h)	0.03 ^c^	0.06 ^b^	0.07 ^a^	0.06 ^a,b^	0.003	0.003	0.016
ED (%)	57.48 ^a^	56.52 ^b^	54.04 ^c^	53.26 ^d^	1.073	<0.001	0.804

BS = budding stage; ES = early flowering stage; PS = peak flowering stage; HS = heading stage. a: rapidly degradable fraction; b: slowly degradable fraction; a + b: total degradable fraction; c: degradation rate of slowly degradable fraction; ED: effective degradability. In the same column, values with different letter mean significant difference (*p* < 0.05).

**Table 3:** Rumen degradability and degradation parameters of CP of amaranth silage.

Items	Growth Stage				SEM	*p*-Value	
	BS	ES	PS	HS		Linear	Quadratic
Time (%)							
4	45.87 ^b^	50.13 ^a^	46.84 ^b^	47.68 ^a,b^	1.185	0.042	0.031
8	50.07 ^c^	57.81 ^b^	57.07 ^b^	61.07 ^a^	0.530	0.056	0.008
16	66.53 ^a^	67.21 ^a^	63.34 ^b^	65.15 ^a,b^	1.415	0.070	0.013
24	69.78	71.29	70.17	71.30	1.818	0.340	0.189
36	73.36 ^b^	74.80 ^a,b^	73.57 ^b^	76.75 ^a^	2.076	0.036	0.310
48	82.13 ^a^	79.08 ^b,c^	80.77 ^a,b^	77.47 ^c^	1.474	0.022	0.213
72	86.02 ^a^	82.14 ^b^	84.97 ^a^	82.97 ^b^	1.227	0.034	0.040
Rumen Degradation Parameters							
a (%)	37.62 ^b^	43.18 ^a^	42.46 ^a^	42.06 ^a^	0.453	0.039	0.042
b (%)	49.86 ^a^	38.87 ^c^	44.61 ^b^	39.90 ^c^	0.801	0.008	0.032
a + b (%)	87.48 ^a^	82.05 ^b^	87.07 ^a^	81.96 ^b^	0.776	0.017	0.024
c (%/h)	0.04 ^a,b^	0.06 ^a^	0.04 ^b^	0.06 ^a^	0.002	0.006	0.007
ED (%)	66.69 ^b^	68.01 ^a^	67.31 ^a,b^	67.95 ^a^	1.079	0.321	0.036

BS = budding stage; ES = early flowering stage; PS = peak flowering stage; HS = heading stage. a: rapidly degradable fraction; b: slowly degradable fraction; a + b: total degradable fraction; c: degradation rate of slowly degradable fraction; ED: effective degradability. In the same column, values with different letter mean significant difference (*p* < 0.05).

**Table 4:** Rumen degradability and degradation parameters of NDF of amaranth silage.

Items	Growth Stage				SEM	*p*-Value	
	BS	ES	PS	HS		Linear	Quadratic
Time (%)							
4	11.67	13.94	12.60	11.44	1.471	0.620	0.248
8	15.40 ^b^	18.18 ^a,b^	21.13 ^a^	20.32 ^a^	0.724	0.044	0.029
16	20.02 ^b^	26.30 ^a^	24.97 ^a^	22.17 ^b^	0.271	0.032	0.016
24	31.54 ^c^	38.19 ^a^	37.46 ^a^	35.29 ^b^	1.339	0.022	0.008
36	37.03	40.88	38.68	37.29	2.851	0.673	0.260
48	51.12 ^a^	45.06 ^b^	49.41 ^a^	46.35 ^b^	1.428	0.040	0.027
72	59.16 ^a^	56.40 ^b^	58.23 ^a^	56.78 ^b^	1.200	0.021	0.033
Rumen Degradation Parameters							
a (%)	5.41	5.87	7.29	6.67	2.010	0.844	0.230
b (%)	70.13 ^a^	45.71 ^b^	40.95 ^c^	42.26 ^b,c^	1.668	<0.001	0.011
a + b (%)	75.54 ^a^	51.58 ^b^	48.24 ^b^	48.93 ^b^	2.413	<0.001	0.018
c (%/h)	0.02 ^b^	0.04 ^a^	0.04 ^a^	0.04 ^a^	0.002	0.003	0.372
ED (%)	31.53 ^a^	32.26 ^a^	30.99 ^a,b^	29.81 ^b^	1.225	0.194	0.016

BS = budding stage; ES = early flowering stage; PS = peak flowering stage; HS = heading stage. a: rapidly degradable fraction; b: slowly degradable fraction; a + b: total degradable fraction; c: degradation rate of slowly degradable fraction; ED: effective degradability. In the same column, values with different letter mean significant difference (*p* < 0.05).

**Table 5:** Rumen degradability and degradation parameters of ADF of amaranth silage.

Items	Growth Stage				SEM	*p*-Value	
	BS	ES	PS	HS		Linear	Quadratic
Time (%)							
4	10.31 ^a^	7.37 ^b^	6.85 ^b^	10.60 ^a^	0.431	0.030	0.004
8	14.56	9.21	14.45	12.00	2.621	0.583	0.109
16	17.41 ^a^	14.32 ^b^	18.04 ^a^	14.82 ^b^	0.517	0.018	0.048
24	32.40 ^a^	26.28 ^c^	29.25 ^b^	29.48 ^b^	1.319	0.036	0.010
36	38.81 ^a^	30.83 ^b^	29.61 ^b^	31.41 ^b^	1.369	0.013	0.021
48	50.62 ^a^	33.68 ^b,c^	32.11 ^c^	35.71 ^b^	1.172	0.018	0.014
72	54.44 ^a^	41.55 ^c^	44.49 ^b^	42.10 ^c^	1.181	0.014	0.039
Rumen Degradation Parameters							
a (%)	3.03 ^a,b^	1.35 ^b^	4.77 ^a^	5.00 ^a^	0.333	0.001	<0.001
b (%)	67.23 ^a^	47.72 ^b^	46.60 ^b^	45.88 ^b^	1.177	<0.001	0.337
a + b (%)	70.26 ^a^	49.07 ^b^	51.37 ^b^	50.88 ^b^	1.583	<0.001	0.016
c (%/h)	0.02	0.03	0.02	0.02	0.007	0.761	0.418
ED (%)	30.84 ^a^	22.90 ^b^	24.65 ^b^	24.83 ^b^	1.119	0.226	0.024

BS = budding stage; ES = early flowering stage; PS = peak flowering stage; HS = heading stage. a: rapidly degradable fraction; b: slowly degradable fraction; a + b: total degradable fraction; c: degradation rate of slowly degradable fraction; ED: effective degradability. In the same column, values with different letter mean significant difference (*p* < 0.05).

**Table 6:** Correlation of amaranth silage between effective degradability and ruminal degradability of DM, CP, NDF, and ADF at different time points.

Items	Regression Equation	R^2^	*p*-Value	Items	Regression Equation	R^2^	*p*-Value
DM				CP			
4 h	y = 0.8074 x + 21.475	0.9242	0.037	4 h	-	-	0.153
8 h	-	-	0.166	8 h	-	-	0.092
16 h	-	-	0.244	16 h	-	-	0.908
24 h	y = 0.8534 x + 4.209	0.9468	0.027	24 h	y = 0.7777 x + 12.559	0.9527	0.024
36 h	y = 0.7142 x + 10.173	0.9606	0.020	36 h	-	-	0.216
48 h	y = 0.4485 x + 25.840	0.9039	0.049	48 h	-	-	0.075
72 h	-	-	0.093	72 h	y = −0.3388 x + 95.955	0.9483	0.026
NDF				ADF			
4 h	-	-	0.236	4 h	-	-	0.395
8 h	-	-	0.447	8 h	-	-	0.308
16 h	-	-	0.618	16 h	-	-	0.440
24 h	-	-	0.843	24 h	-	-	0.067
36 h	-	-	0.327	36 h	-	-	0.053
48 h	-	-	0.989	48 h	y = 0.3907 x + 10.945	0.9220	0.040
72 h	-	-	0.935	72 h	y = 0.5644 x + 0.042	0.9539	0.023

x represents rumen degradability at different time point; y represents effective degradability. “-” represents no significant correlation.

## Supplementary Materials

The following are available online at https://www.mdpi.com/2076-2615/9/10/793/s1, Figure S1: Fermentation container.
