# Supplementary material for: Effects of Different Growth Stages of Amaranth Silage on the Rumen Degradation of Dairy Cows
Source: Animals (Basel). 2019 Oct 12;9(10):793. doi: 10.3390/ani9100793 (PMC6826743; doi:10.3390/ani9100793)
Supplement: Supplementary file 1 [file animals-09-00793-s001.pdf]

Effects of different growth stages of amaranth silage on the rumen degradation of dairy cows

Jian Ma[1,3], Guoqing Sun[1,2#], Ali Mujtaba Shah[3,4], Xue Fan[1], Shengli Li[2*] and Xiong Yu[1*]

[1] College of Animal Science, Xinjiang Agricultural University, Urumchi 100193, China; Crazyma0411@163.com (J.M.); jackjeons123@163.com (G.S.); fanxue1205@163.com (X.F.)
[2] College of Animal Science, China Agricultural University, Beijing 830052, China;
[3] Animal Nutrition Institute, Sichuan Agricultural University, Chengdu 611130, China alimujtabashah@sbbuvas.edu.pk (A.M.S.)
[4] Department of Livestock Production, Shaheed Benazir Bhutto University of Veterinary and Animal Sciences, Sakrand 67210 Sindh Pakistan
[*] Correspondence: lishengli@cau.edu.cn (S.L.); yuxiong8763601@126.com (X.Y.); Tel.: +86-10-13331168629 (S.L.); +86-991-15276763575 (X.Y.)
Jian Ma and Guoqing Sun contributed equally to this work

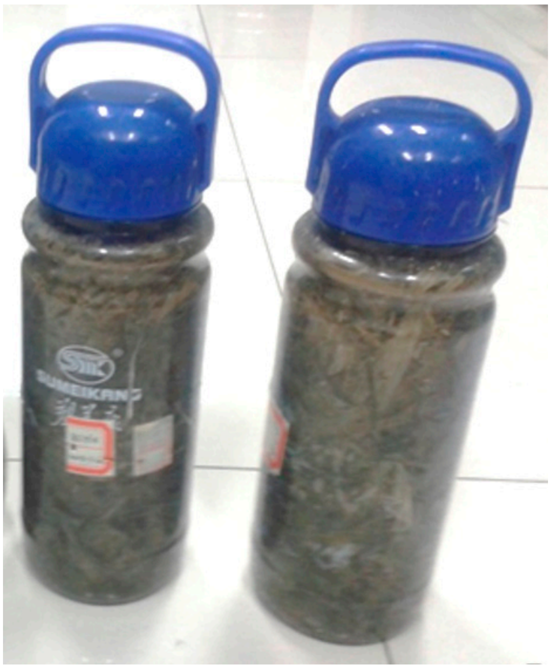

Figure S1. Fermentation container.
